# A hybrid deep learning scheme for MRI-based preliminary multiclassification diagnosis of primary brain tumors

**DOI:** 10.3389/fonc.2024.1363756

**Published:** 2024-04-30

**Authors:** Zhichao Wang, Chuchu He, Yan Hu, Haifeng Luo, Chao Li, Xiandong Wu, Yang Zhang, Jingjing Li, Jun Cai

**Affiliations:** ^1^ Department of Oncology, The First Affiliated Hospital of Yangtze University, Jingzhou, Hubei, China; ^2^ Hubei Key Laboratory of Precision Radiation Oncology, Wuhan, China

**Keywords:** brain tumor classification, MRI images, deep learning, transfer learning, model interpretability

## Abstract

**Objectives:**

The diagnosis and treatment of brain tumors have greatly benefited from extensive research in traditional radiomics, leading to improved efficiency for clinicians. With the rapid development of cutting-edge technologies, especially deep learning, further improvements in accuracy and automation are expected. In this study, we explored a hybrid deep learning scheme that integrates several advanced techniques to achieve reliable diagnosis of primary brain tumors with enhanced classification performance and interpretability.

**Methods:**

This study retrospectively included 230 patients with primary brain tumors, including 97 meningiomas, 66 gliomas and 67 pituitary tumors, from the First Affiliated Hospital of Yangtze University. The effectiveness of the proposed scheme was validated by the included data and a commonly used data. Based on super-resolution reconstruction and dynamic learning rate annealing strategies, we compared the classification results of several deep learning models. The multi-classification performance was further improved by combining feature transfer and machine learning. Classification performance metrics included accuracy (ACC), area under the curve (AUC), sensitivity (SEN), and specificity (SPE).

**Results:**

In the deep learning tests conducted on two datasets, the DenseNet121 model achieved the highest classification performance, with five-test accuracies of 0.989 ± 0.006 and 0.967 ± 0.013, and AUCs of 0.999 ± 0.001 and 0.994 ± 0.005, respectively. In the hybrid deep learning tests, LightGBM, a promising classifier, achieved accuracies of 0.989 and 0.984, which were improved from the original deep learning scheme of 0.987 and 0.965. Sensitivities for both datasets were 0.985, specificities were 0.988 and 0.984, respectively, and relatively desirable receiver operating characteristic (ROC) curves were obtained. In addition, model visualization studies further verified the reliability and interpretability of the results.

**Conclusions:**

These results illustrated that deep learning models combining several advanced technologies can reliably improve the performance, automation, and interpretability of primary brain tumor diagnosis, which is crucial for further brain tumor diagnostic research and individualized treatment.

## 1 Introduction

Brain and central nervous system (CNS) tumors are among the most deadly cancers and have a high incidence. In the United States, approximately 80,000 people were diagnosed with brain or CNS tumors in 2021, and 18,600 died from these diseases (1). Common brain tumors include gliomas, meningiomas, and pituitary tumors (approximately 23%, 38%, and 17% of primary brain and CNS tumors, respectively) (2, 3). Primary intracranial tumors arise from various sites, including brain tissue, meninges, pituitary gland, cranial nerves, and vascular tissue. Available treatment options include surgical resection, radiotherapy, and chemotherapy. Therefore, accurate early diagnosis is essential for individualized treatment and prognostic assessment.

Magnetic resonance imaging (MRI) is a widely employed technique for the preliminary diagnosis of brain tumors, which can provide clear visualization of the nervous system structure and local lesions. Clinical application of MRI-based manual diagnosis can be influenced by professional level, work pressure, and degree of automation. In recent years, artificial intelligence has achieved significant progress in medicine (4, 5). Numerous studies have investigated the potential of machine learning combined with radiomics in brain tumor detection, molecular and genetic diagnosis (6–8). Nevertheless, further improvements are needed regarding the level of automation, reproducibility, and feature extraction performance of machine learning in radiomics to address the limitations associated with its inherent flaws (9–11).

As deep learning has demonstrated powerful adaptive feature extraction and end-to-end advantages in various fields, intelligent tumor diagnosis has also been widely researched and clinical application. Afshar analyzed the advantages of deep learning-based radiomics, such as freedom from prior knowledge and target area outlining, and end-to-end training (11). Lao validated the potential of deep learning in feature extraction and overall survival prediction based on 112 glioma patients (12). However, while acknowledging the significance of preliminary brain tumors diagnosis, the recent report highlighted the presence of the Smart Hans phenomenon in automated classification studies, where the model achieved better results without specifically focusing on the tumor region (13). This study revealed this previously overlooked bias, and provided valuable guidance for subsequent research. On one hand, deep learning models integrating multiple cutting-edge technologies can adaptively extract local information and assign appropriate weights, showing better performance than undifferentiated manual feature extraction based on the entire slice. On the other hand, the model visualization is crucial for interpreting whether it indeed focuses on the tumor area, which is an important verification of diagnostic reliability.

Therefore, this study proposed a hybrid deep learning scheme that integrated several advanced technologies for automated preliminary diagnosis of tumors. By focusing on the study of primary brain tumors, this study provided an important foundation for further extensions, such as brain metastasis prediction and pathological classification. The main contributions of this work are summarized as follows:

- In model construction, super-resolution reconstruction and dynamic learning rate strategies were applied to improve image quality and training efficiency.
- Based on the advantages of deep learning (DL) in feature extraction, machine learning models were further combined to improve classification performance.
- To assess the generalization performance and alleviate the interpretability problem, this study utilized t-SNE and Score-Grad techniques and verified the effectiveness of the scheme based on our institute and public datasets.

The remaining parts are organized as follows. Section 2 (Materials and methods) introduces the patient population and methodologies employed in the proposed scheme. The datasets and results of the experiments are presented in Section 3 (Results). Based on the displayed results, Section 4 (Discussion) analyzes the performance of the proposed scheme in detail. Finally, conclusions are summarized in Section 5 (Conclusions).

## 2 Materials and methods

### 2.1 Patient population

This retrospective study was approved by the ethics committees of the First Affiliated Hospital of Yangtze University, and informed consent was waived. Patients were enrolled based on the following criteria: 1) available postoperative pathological diagnosis results; 2) MRI examinations performed in our hospital within 2 weeks before surgery; 3) available medical records. Exclusion criteria were as follows: 1) history of preoperative treatment (radiation, chemotherapy, or other treatments); 2) unavailable contrast-enhanced T1-weighted sequence; 3) presence of MRI artifacts or tumors too small to seriously affect tumor imaging.

### 2.2 Image acquisition and preprocessing

Contrast-enhanced MRI scans were performed at our institution using two 1.5 T scanners (Philips prodiva) and one 3.0 T scanner. MRI examinations included T1-weighted imaging (T1WI), T2-weighted imaging (T2WI), fluid-attenuated inversion recovery (Flair), diffusion-weighted imaging (DWI), and contrast-enhanced T1WI (CE-T1WI). The CE-T1WI sequence included transverse, sagittal, and coronal views, and several slices near the largest tumor level were acquired in each view. In addition, multi-sequence modal analysis can be considered in further studies. The scan parameter settings were as follows: a repetition time (TR) and echo time (TE) of 5.5ms and 2.4ms, a pixel matrix of 256*236, a slice thickness and slice gap of 1mm and 0.5mm, and a deflection angle of 15 degrees.

**Figure 1:**
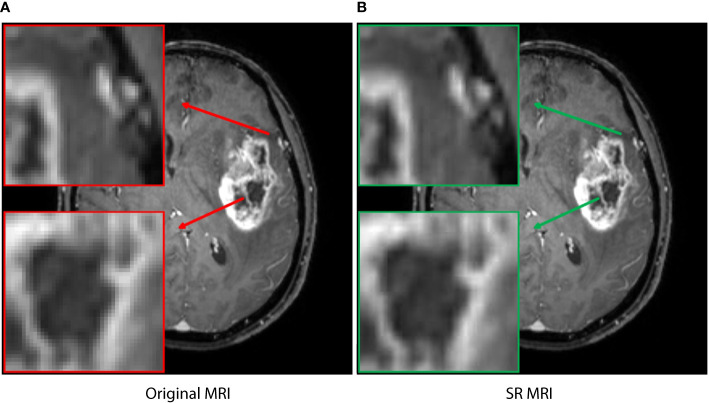
The super-resolution reconstruction result based on a generative adversarial network model. The reconstructed image **(B)** is not only very similar to the original transverse image **(A)**, but also has more reasonable edges and finer textures. The boxes represent local enlarged images, with red and green arrows representing the original MRI and SR MRI, respectively.

In addition, image resolution can be affected by factors such as hardware configuration, acquisition time, and radiation exposure, which can potentially hinder accurate diagnosis and treatment. In recent years, super-resolution reconstruction technology based on artificial intelligence has been widely studied in image preprocessing and data enhancement, with strong evidence of its effectiveness (14–17). In this study, the generative adversarial network (GAN) model supported by the Onekey platform was applied to learn the mapping from low-resolution to high-resolution, thereby improving the spatial resolution of the MRI slice in detail (18). The model was trained on millions of medical images, which enabled high-quality image preprocessing, including denoising, artifact removal, and intensity values normalization. The resolution of MRI slice was increased by a factor of 4, resulting in a transformation from 1*1 pixels to 0.25*0.25 (**Figure 1**). Although the image enhancement was reflected in small pixel changes, it provided the deep learning model with accurate feature information and fine tumor boundaries.

### 2.3 Deep learning model construction

**Figure 2:**
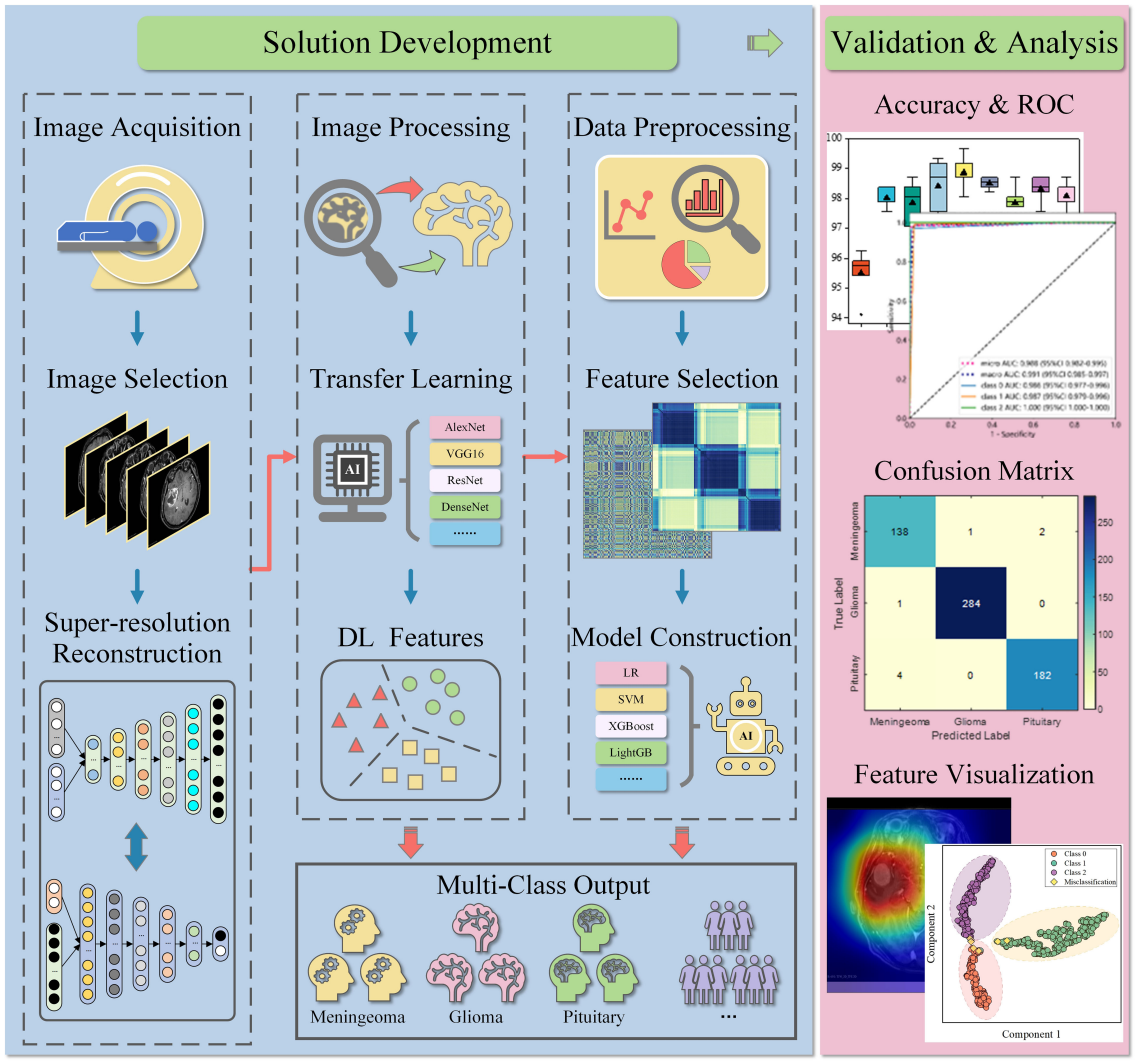
Workflow of the study. MRIs were retrospectively collected and selected, then pre-processed and input into the deep learning model. In deep learning diagnosis, the model can directly output common tumor types. In the hybrid classification scheme, the extracted DL features were further used for machine learning construction after preprocessing and selection. The performance of both schemes was verified and analyzed on the test set. LR, logistic regression; SVM, support vector machines; XGBoost, eXtreme gradient boosting; LightGB, light gradient boosting machine.

The workflow of this study is illustrated in **Figure 2**. After image acquisition and preprocessing, the model construction was employed. This model consisted of a deep learning network, a feature adaptation module, and a full-connected classification layer. The deep learning networks were pre-trained using real images, including AlexNet, VGG16, ResNet18, ResNet50, DenseNet121, DenseNet169, GoogleNet, MobileNetV2, and MobileNetV3. The feature adaptation module consisted of two fully-connected layers and was connected behind the deep learning network. Its output was called deep learning (DL) feature with a dimension of 128. The DL features were then input into the classification layer to obtain the final tumor type. 5-fold cross-validation was implemented in this work to avoid overfitting of the deep learning model and ensure the reliability of the results.

Model training is critical to classification results and involves fine-tuning of hyperparameters, especially the learning rate. A fixed learning rate can lead to non-convergence or a local optimal solution. Inspired by recent successful applications of various dynamic learning rate strategies, this study applied them to improve model training efficiency and classification performance (19–22). It is recommended to adopt a larger learning rate in early training and reduce the learning rate with iteration. Therefore, based on the results of several previous tests, the dynamic learning annealing rate of *lr* = 0.01/(1 + 10 * *p)* ^0.75^ was applied in model training. The *p* changed linearly from 0 to 1 with iteration. The batch size and momentum were set to 32 and 0.9, and models were trained for 200 epochs. During model training, cross-entropy loss was calculated, parameters were optimized based on stochastic gradient descent algorithm and backpropagation algorithm.

### 2.4 Deep learning feature and machine learning construction

As shown in **Figure 2**, the hybrid scheme integrates deep learning features and machine learning to improve classification accuracy. In this study, 128-dimensional DL features were first applied for data preprocessing, including data format validation, statistical outlier detection, and z-score standardization. Subsequently, Pearson’s correlation coefficient was calculated for preliminary feature evaluation and selection, with the threshold set at 0.9. Features were filtered in the training set based on the least absolute shrinkage and selection operator (LASSO), and non-zero items in high-dimensional features were determined as available inputs. Ultimately, non-redundant low-dimensional features can be used to construct machine learning classifier, and perform preliminary diagnose of brain tumors.

### 2.5 Model performance evaluation

In the deep learning scheme, various key metrics such as accuracy, AUC, sensitivity and specificity were calculated to evaluate the classification performance. The multi-classification performance was demonstrated based on the confusion matrix. More importantly, feature visualization was performed to explore the reliability and interpretability of the model. In the hybrid scheme, accuracy, sensitivity and specificity were used to compare the classification performance of machine learning, including LR, NaiveBayes, SVM, RandomForest, ExtraTrees, XGBoost, LightGBM, Adaptive Boosting (AdaBoost), Multi-layer Perceptron (MLP). The ROC curves were compared using the DeLong test to analyze multi-classification performance.

### 2.6 Statistical analysis

Statistical analysis was performed using SPSS (version 26.0). Continuous variables were described as mean ± standard deviation, while categorical variables were presented as frequencies and percentages. Continuous variables were analyzed using Student’s t test or analysis of variance. Chi-square test or Fisher’s exact test was used to compare categorical variables. P value < 0.05 was considered statistical significance. Data preprocessing and feature evaluation, LASSO regression analysis, and DeLong test were performed using Python (version 3.11).

## 3 Results

### 3.1 General patient characteristics

Between January 2018 and December 2022, a total of 230 patients with common brain tumors were enrolled in this study. They were initially divided into meningiomas (97 cases), gliomas (66 cases), and pituitary tumors (67 cases) according to the pathological results, which were labeled as 0, 1, 2 in this study. This dataset was labeled as BT-YU in this study. In one data division of 5-fold cross-validation, the baseline clinical characteristics in the training and test cohorts were presented in **Table 1**. These characteristics included histologic diagnosis and demographic information. No significant differences were observed in any of the detailed characteristics between the two cohorts (all P > 0.05).

**Table 1:** Patient characteristics.

Characteristics	Entire Cohort (n=230)	Training Cohort (n=185)	Test Cohort (n=45)	*P* value
**Age, year**	54.63 ± 12.53	54.79 ± 12.61	53.96 ± 12.32	0.820
**Tumor size, cm**	4.17 ± 1.60	4.13 ± 1.57	4.34 ± 1.78	0.233
**Gender, No. (%)**				0.309
Male	92	77	15	
Female	138	108	30	
**Tumor position (%)**				0.271
Frontal Lobe	78	64	14	
Parietial Lobe	20	19	1	
Occipital Lobe	7	4	3	
Temporal & Insular Lobe	40	29	11	
cerebellum	15	12	3	
pituitary	67	54	13	
Others	3	3	0	
**MRI Images**	2901	2334	567	

### 3.2 The performance of various deep learning models

**Figure 3:**
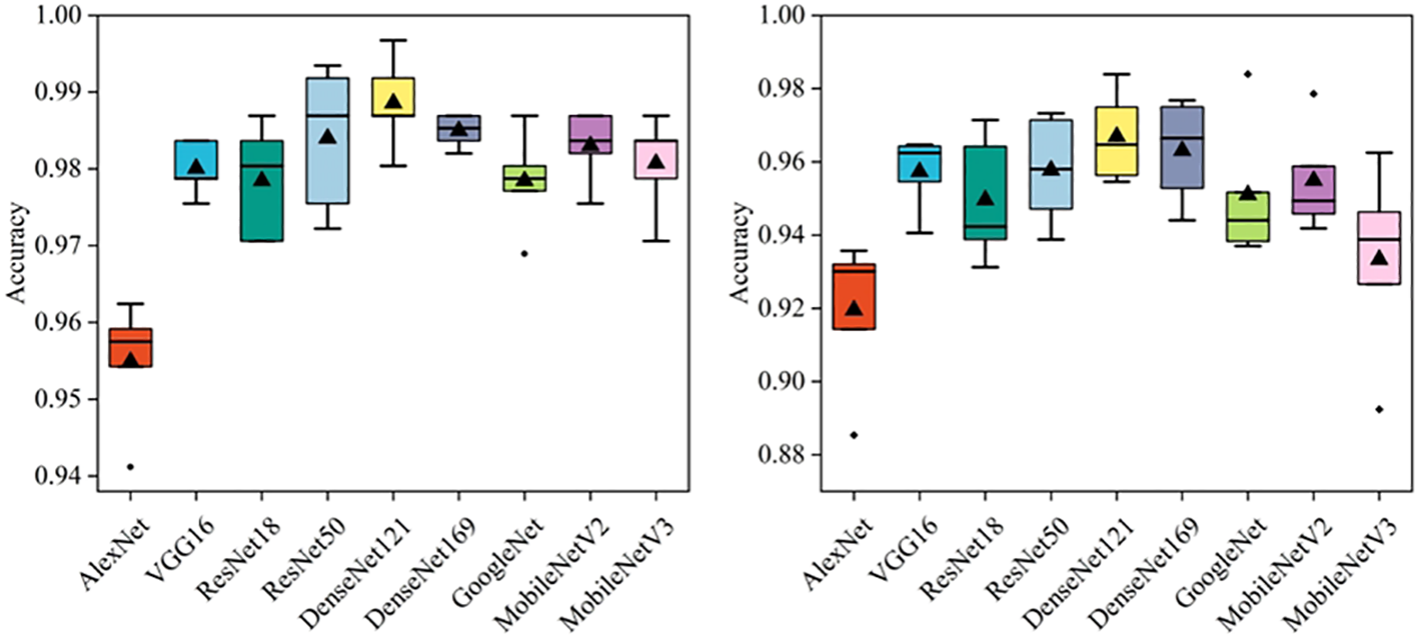
The diagnostic results of deep learning models in five tests.

In this section, several deep learning models were compared, including AlexNet, VGG16, ResNet18, ResNet50, DenseNet121, DenseNet169, GoogleNet, MobileNetV2, and MobileNetV3. Besides, a commonly used dataset CE-MRI was applied as an auxiliary test to further verify the effectiveness of the models (23). CE-MRI is a T1-weighted contrast-enhanced MRI image set with a total of 3064 images, including meningiomas (708 slices), gliomas (1426 slices) and pituitary tumors (930 slices). The images are a combination of transverse, sagittal and coronal, with a resolution of 512 × 512. The classification of CE-MRI was consistent with the BT-YU dataset. In the 5-fold cross-validation test, the influence of random effects and overfitting were avoided, and the results are shown in **Figure 3**; **Table 2**. Further, **Supplementary Tables S1, S2** show the detail classification performance. **Table 2** also presents the results of state-of-the-art models on the CE-MRI dataset. Among them, references (23) and (27) represent methods combining radiomics and machine learning, references (24) (25), and (26) represent cutting-edge convolutional neural network (CNN) models, and reference (28) represents an improved vision transformer model.

**Table 2:** The diagnostic results of deep learning models.

Datasets	Models	ACC	AUC	SEN	SPE
CE-MRI	AlexNet	0.955 ± 0.008	0.994 ± 0.002	0.949 ± 0.007	0.953 ± 0.008
	VGG16	0.980 ± 0.004	0.997 ± 0.002	0.979 ± 0.005	0.980 ± 0.004
	ResNet18	0.978 ± 0.008	0.999 ± 0.001	0.976 ± 0.008	0.977 ± 0.008
	ResNet50	0.984 ± 0.010	0.998 ± 0.002	0.981 ± 0.011	0.983 ± 0.010
	DenseNet121	0.989 ± 0.006	0.999 ± 0.001	0.987 ± 0.007	0.988 ± 0.006
	DenseNet169	0.985 ± 0.002	0.999 ± 0.001	0.983 ± 0.003	0.984 ± 0.002
	GoogleNet	0.978 ± 0.007	0.998 ± 0.001	0.975 ± 0.008	0.978 ± 0.007
	MobileNetV2	0.983 ± 0.005	0.999 ± 0.000	0.982 ± 0.006	0.983 ± 0.005
	MobileNetV3	0.981 ± 0.006	0.998 ± 0.001	0.978 ± 0.007	0.980 ± 0.007
	Radiomics (23)	0.9128	——	——	——
	CNN (24)	0.9780	0.9890	0.9640	0.9830
	VGG16 (25)	0.9800	0.9900	0.9800	0.9800
	CNN (26)	0.9870	——	0.9860	0.9870
	BoF-SURF + KNN (27)	0.9870	——	0.9840	0.9860
	RanMerFormer (28)	0.9886	——	0.9846	0.9939
BT-YU	AlexNet	0.920 ± 0.021	0.979 ± 0.009	0.914 ± 0.031	0.918 ± 0.022
	VGG16	0.957 ± 0.010	0.994 ± 0.004	0.958 ± 0.012	0.957 ± 0.011
	ResNet18	0.950 ± 0.017	0.990 ± 0.005	0.947 ± 0.020	0.949 ± 0.018
	ResNet50	0.958 ± 0.015	0.993 ± 0.006	0.957 ± 0.020	0.957 ± 0.016
	DenseNet121	0.967 ± 0.013	0.994 ± 0.005	0.966 ± 0.014	0.967 ± 0.013
	DenseNet169	0.963 ± 0.014	0.996 ± 0.003	0.963 ± 0.014	0.963 ± 0.014
	GoogleNet	0.951 ± 0.019	0.992 ± 0.006	0.948 ± 0.023	0.950 ± 0.020
	MobileNetV2	0.955 ± 0.015	0.994 ± 0.003	0.952 ± 0.019	0.954 ± 0.015
	MobileNetV3	0.933 ± 0.026	0.985 ± 0.013	0.930 ± 0.037	0.933 ± 0.028

- indicates that the indicator is not included in the listed literature.

**Figure 3** visually shows the results of the five tests. The DenseNet121 model achieved the highest accuracy and relatively low standard deviation in both datasets. In addition, **Table 2** quantitatively describes the average statistical indicators of the tests. The classification performance of the DenseNet121 model reached the optimal level, with an accuracy of 0.989 ± 0.006, AUC of 0.999 ± 0.001, sensitivity and specificity of 0.987 ± 0.007 and 0.988 ± 0.006 in CE-MRI; the accuracy in BT-YU was 0.967 ± 0.013, AUC was 0.994 ± 0.005, sensitivity and specificity were 0.966 ± 0.014 and 0.967 ± 0.013, respectively.

**Figure 4:**
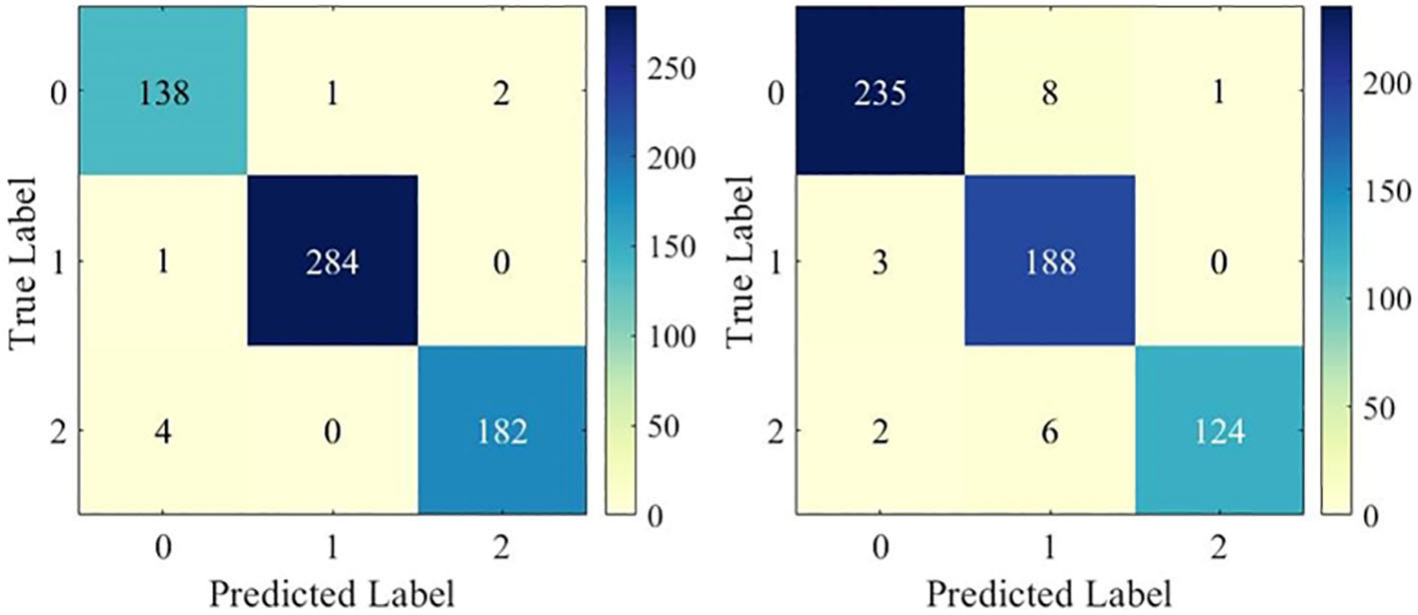
Confusion matrix of DenseNet121 model.

To examine the multi-classification performance in detail, **Figure 4** shows the confusion matrix of the DenseNet121 model in one test. It can be observed that the model showed excellent multi-classification performance for brain tumors. However, as shown in **Table 2**, the metrics of DenseNet121 model were not all optimal. For example, its AUC on BT-YU was slightly lower than that of the DenseNet169 model. Therefore, actual applications require model selection based on specific conditions.

### 3.3 The performance of various hybrid models

**Figure 5:**
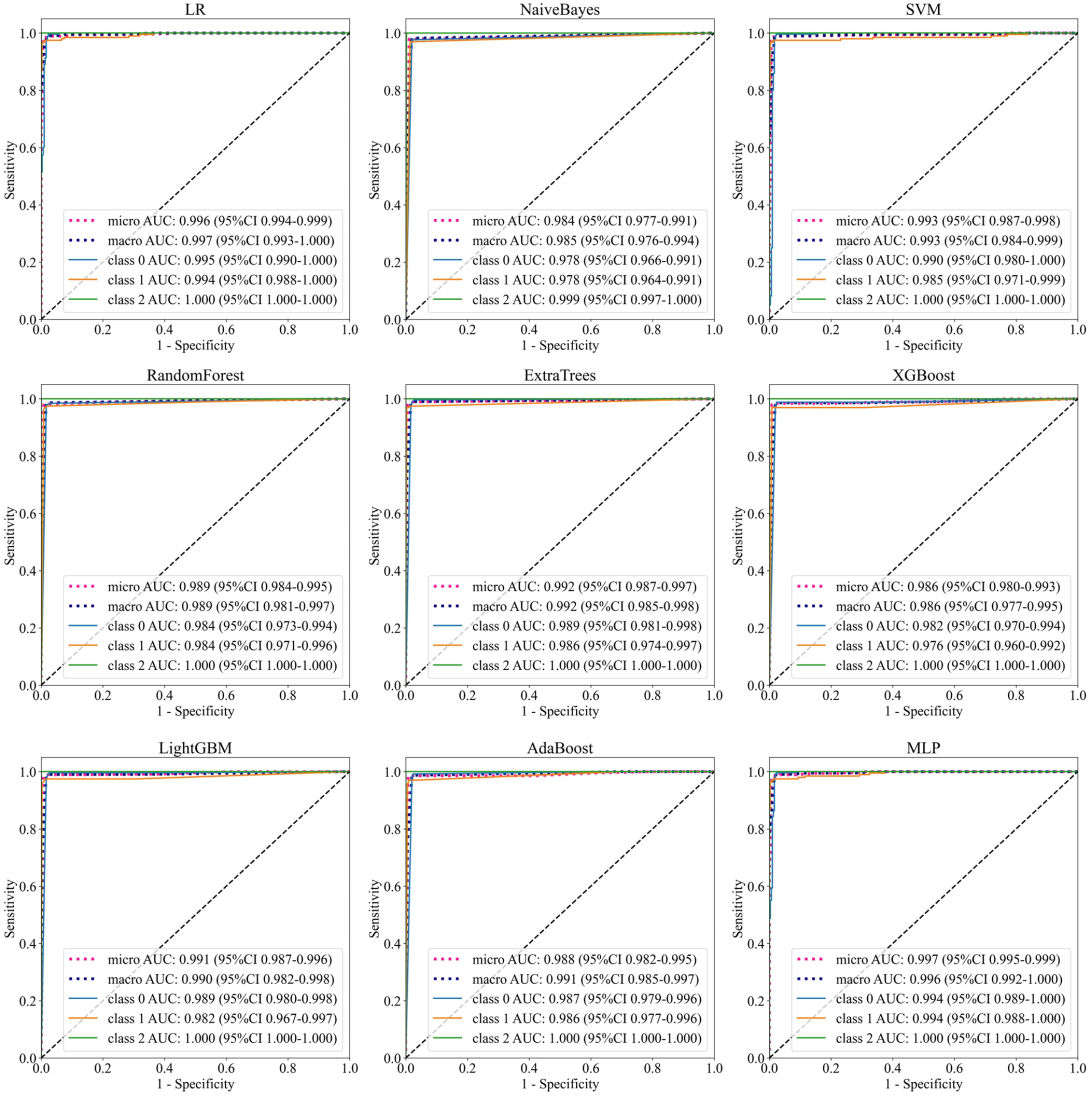
ROC curves of machine learning models on BT-YU dataset.

To further improve accuracy of the Densenet121 model, this section combined deep learning features and machine learning classifiers for diagnostic testing. The models include LR, NaiveBayes, SVM, RandomForest, ExtraTrees, XGBoost, LightGBM, AdaBoost, and MLP. After feature selection and preprocessing, the 128-dimensional deep learning features were applied to construct machine learning classifier. **Table 3** shows the learning effect of the model on the deep learning features in one test. In addition, **Figure 5** shows the multi-classification ROC curve of each model in the BT-YU dataset. In summary, the LightGBM model maintained the highest accuracy on both datasets, which were 0.989 and 0.984 respectively. Compared to the original Densenet121 model, the accuracy of this hybrid scheme was improved to some extent, especially on the BT-YU dataset. The AUC of the LightGBM model was slightly lower than the original Densenet121 model, but the sensitivity and specificity were optimal (0.985 and 0.988 in CE-MRI dataset; 0.985 and 0.984 in BT-YU dataset). Further, **Supplementary Tables S3, S4** show the detail classification performance.

**Table 3:** The diagnostic results of machine learning models.

Datasets	Models	ACC	AUC	SEN	SPE
CE-MRI	—^*^	0.987	0.997	0.985	0.986
	LR	0.987	0.996	0.983	0.986
	NaiveBayes	0.786	0.794	0.692	0.764
	SVM	0.987	0.996	0.983	0.986
	RandomForest	0.982	0.994	0.978	0.981
	ExtraTrees	0.985	0.994	0.982	0.984
	XGBoost	0.985	0.993	0.981	0.984
	LightGBM	0.989	0.993	0.985	0.988
	AdaBoost	0.987	0.995	0.984	0.986
	MLP	0.984	0.996	0.981	0.983
BT-YU	—^*^	0.965	0.985	0.962	0.964
	LR	0.980	0.996	0.983	0.981
	NaiveBayes	0.979	0.985	0.982	0.979
	SVM	0.980	0.992	0.983	0.981
	RandomForest	0.980	0.989	0.983	0.981
	ExtraTrees	0.982	0.992	0.985	0.982
	XGBoost	0.982	0.986	0.984	0.982
	LightGBM	0.984	0.990	0.985	0.984
	AdaBoost	0.982	0.991	0.985	0.982
	MLP	0.982	0.996	0.985	0.982

*: The horizontal line indicates the results of the original DenseNet21 model in this test.

### 3.4 Feature and model visualization

**Figure 6:**
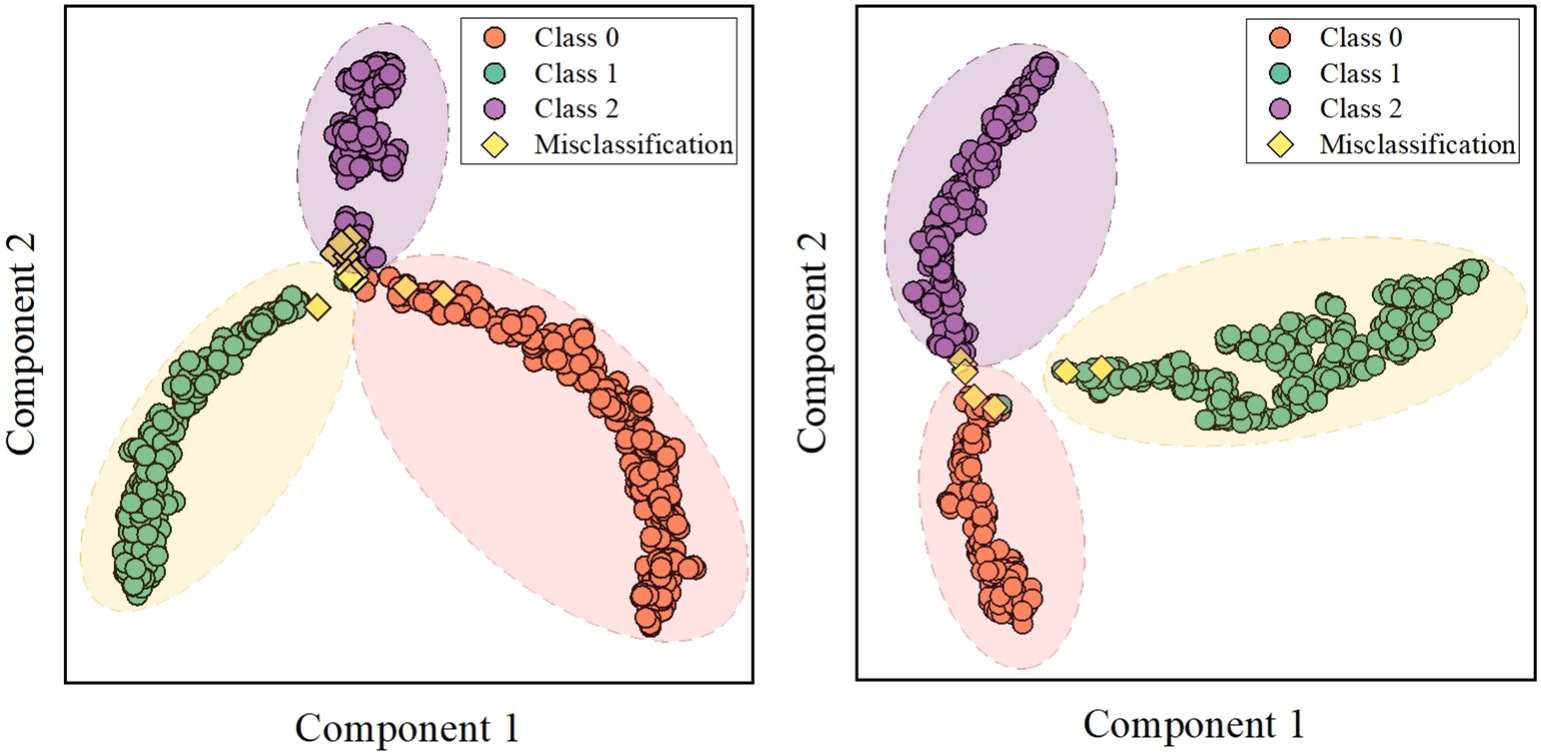
Feature visualization of DenseNet121 model based on t-SNE.

The classification performance of brain tumors in this study depends on the adaptive extraction of features from MRI images. To improve the interpretability of the classification results, this section first implemented feature visualization based on test sets. T-distributed stochastic neighbor embedding (t-SNE) technology can effectively preserve data similarity and local structure information during dimensionality reduction, while also alleviating the congestion problem (29–31). T-SNE was applied for dimensionality reduction of 128-dimensional features of DenseNet121 model, and cluster analysis was performed in 2-dimensional space for intuitive visualization (**Figure 6**). There were several misclassified features distributed in concentrated areas of the three clusters. More importantly, the deep learning features in both datasets were intuitively distinguished, providing useful features for multi-classification.

**Figure 7:**
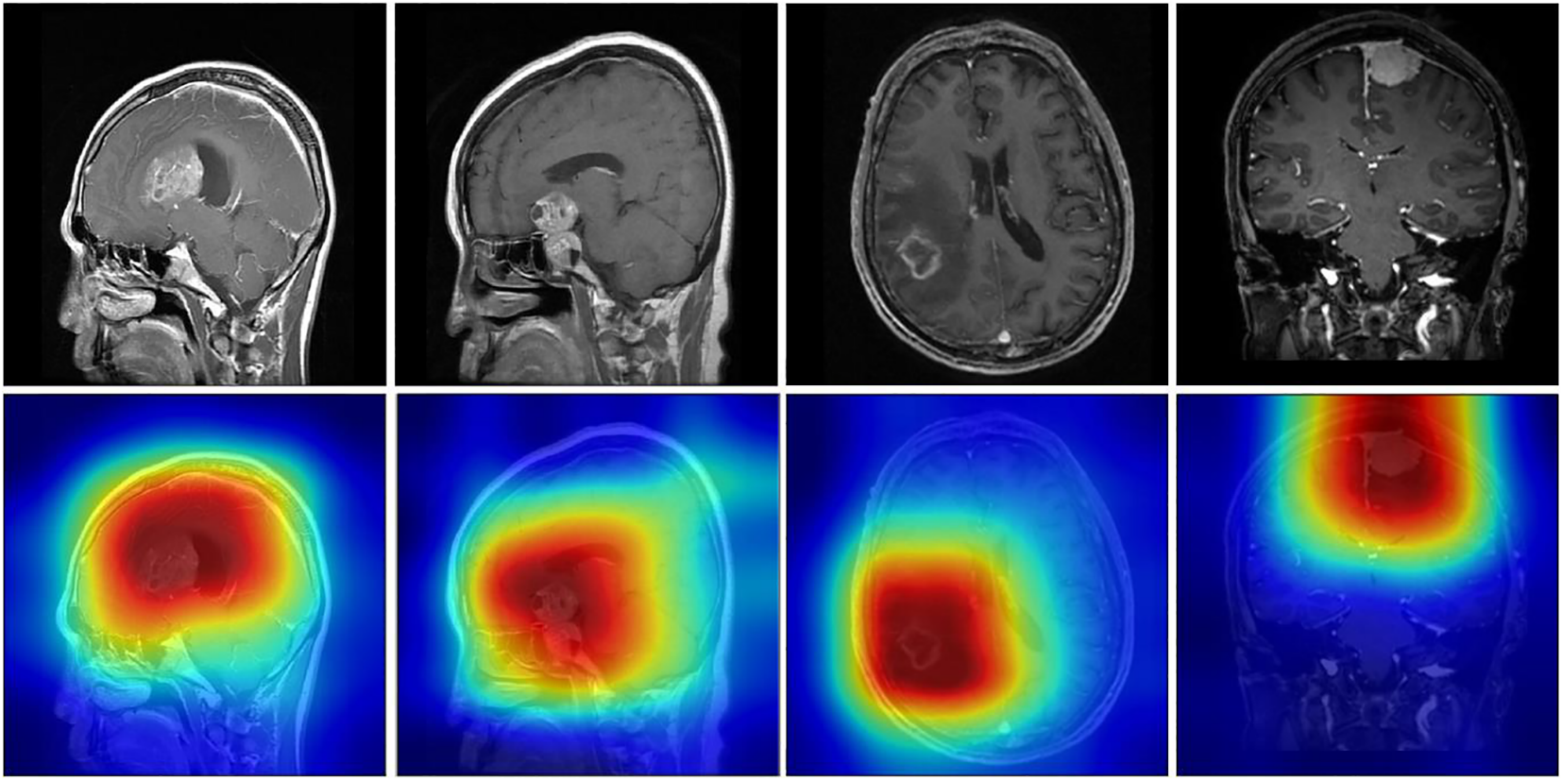
Model attention heatmaps on representative samples.

Score-weighted class activation mapping (Score-CAM) is a model visualization technology that obtains weights by the forward pass score of each activation map on the target class. Score-CAM effectively reduces gradient dependence, thus providing excellent visual representation (32–34). Therefore, Score-CAM was introduced to visualize the attention weights of the DenseNet121 model to demonstrate decision support for classification. **Figure 7** shows representative sample heatmaps in the two datasets. The red core area indicates a large weight area that contributes significantly to the model classification. Besides, this area matched well with the tumor area, which further confirmed the effectiveness of feature extraction.

## 4 Discussion

In this study, we proposed a hybrid intelligent scheme that combined deep learning and machine learning for automated diagnosis of brain tumor types based on CE-MRI images. This solution integrated several advanced technologies to improve feature extraction performance, such as super-resolution reconstruction, dynamic learning rate strategy, convolutional neural network and machine learning. Our proposed scheme demonstrated excellent accuracy, AUC, sensitivity, and specificity. Besides, the generalization performance, robustness, and interpretability of the hybrid scheme were further verified on two datasets.

Brain tumor diagnosis has always been a meaningful and challenging clinical research hotspot. Previous studies have focused on traditional radiomics solutions for predicting glioblastoma, brain metastasis, and isocitrate dehydrogenase (IDH) mutations (35–37). The process of traditional radiomics includes image acquisition, reconstruction and preprocessing, region of interest delineation, manual feature extraction, feature selection, machine learning construction. Meißner developed a radiomics classifier to predict intracranial BRAF V600E mutation status in patients with melanoma brain metastases, and achieved an AUC value of 0.92 (38). Zhao implemented the World Health Organization (WHO) classification of meningiomas based on radiomics and clinical information, and the AUC value reached 0.860 (95% CI, 0.788–0.923) (39). However, recent research has revealed that traditional radiomics may have inherent limitations that restrict its application in clinical and more complex tasks (40, 41). The level of automation for ROI delineation remains a challenge, and reproducibility may be difficult to ensure in testing and prospective applications, leading to low generalization performance. In addition, manual feature extraction is difficult to comprehensively analyze image features, making the test results potentially accidental. Humphries developed a deep learning algorithm using full-resolution axial images as input to diagnose emphysema patterns, which provided important guidance for other tumor diagnosis (42). Therefore, comprehensive analysis of tumor-related slices, as well as adaptive extraction of tumor region features without pre-definition, may achieve more efficient data processing and more robust information mining.

Deep learning has powerful image processing and feature extraction capabilities, which provides an effective technical support for the above limitations. Convolutional neural networks, with many image processing advantages such as local receptive field, weight sharing and down-sampling, are able to extract local and key features, and are widely used in computer vision and target detection (43–46). Bhattacharjee implemented automatic multi-classification diagnosis of full-slice lung and kidney CT images based on the Xception model, and achieved accuracies of 99.39% and 100% respectively (47). Ziegelmayer verified the excellent robustness of deep learning features relative to radiomics features based on CT scans of 60 patients with hepatocellular carcinoma and hepatocolon cancer metastasis (48). Similar to these studies, our deep learning-based model showed good performance in tumor region detection and feature extraction. In addition, the combination of deep learning features and machine learning is considered an effective strategy to further improve the accuracy.

Therefore, to improve the limitations of traditional radiomics, this study first introduced several deep learning models to directly analyze MRI images. These models include AlexNet, VGG16, ResNet18, ResNet50, DenseNet121, DenseNet169, GoogleNet, MobileNetV2, and MobileNetV3. In addition, the proposed solution integrated super-resolution reconstruction technology and dynamic learning rate annealing technology to ensure the quality of image preprocessing and model training.

In the deep learning model test, the DenseNet121 model achieved the best comprehensive classification performance, and its accuracy reached 0.989 ± 0.006 and 0.967 ± 0.013 in the two datasets. It can be seen from **Table 2** that in terms of accuracy, this work was slightly better than the comparison models, which may be caused by the comprehensive factors of image preprocessing, training strategy and model construction. Of course, the accuracy of most models was also close to 0.99, and the results of VGG16, ResNet, GoogleNet and MobileNet models in this work were not significantly lower, which indicated that accuracy, model complexity and training time should be comprehensively considered in model selection. This also inspired us to look for another effective solution to improve accuracy instead of changing deep learning model.

To further improve the accuracy of diagnosis, this study developed a hybrid deep learning scheme for multi-classification of brain tumors. This hybrid scheme essentially applied machine learning to replace the final classification layer of the deep learning model, thus combining the advantages of deep feature extraction and machine learning classification. The machine learning models include LR, NaiveBayes, SVM, RandomForest, ExtraTrees, XGBoost, LightGBM, AdaBoost, and MLP. In the test based on the features extracted by DenseNet121 model, the LightGBM model, as a promising machine learning model, achieved an accuracy of 0.989 and 0.984, a sensitivity of 0.985, and a specificity of 0.988 and 0.984 in the two datasets. Although the AUC of the LightGBM model was not optimal, its ROC curves shown in **Figure 5** were relatively ideal. Therefore, this model was considered to have the best comprehensive multi-class performance. Since the original accuracy of the CE-MRI dataset was already high, the enhancement effect of the hybrid scheme was more obvious in the BT-YU dataset.

In addition, this study utilized the entire MRI as input to improve automation, and it was crucial to explore feature visualization and model focus areas. Therefore, T-SNE and Score-Grad technologies were employed in the visualization research. **Figure 6** shows the 2-dimensional clustering of test sample features, which well illustrates the difference in extracted features between different tumors. **Figure 7** displays the Score-Grad heat map of representative samples, indicating that the focus areas of the model are well consistent with the tumor and peritumoral areas, thus reliably explaining the classification results. In summary, this hybrid scheme can focus on key areas of brain tumors and extract pivotal features, thereby providing decision support for targeted diagnosis and treatment plans.

Of course, we are also aware that various current imaging approaches can be used to non-invasively identify brain tumors (35, 36, 39). In addition, there are many classifications and subtypes of brain tumors, such as brain metastases, gliomas, and meningiomas. It is extremely challenging to develop a single validated solution to diagnose all classifications. Instead, the purpose of this study is not to provide the only effective method, but to demonstrate the potential application value of deep learning in automatic processing and interpretability, and to verify the feasibility of improving accuracy through deep learning and machine learning. Based on our results, the next step is to solve the problem of multi-center validation and detailed classification of brain tumors, such as single brain metastases, glioma subtypes, meningioma grading.

## 5 Conclusions

This study investigated a hybrid deep learning scheme for automated primary brain tumors diagnosis. The scheme integrated various advanced technologies and used two datasets to verify the classification performance and interpretability. By combining super-resolution reconstruction and dynamic learning rate annealing technologies, the deep learning model achieved high classification accuracy. Furthermore, based on deep feature transfer and machine learning models, the performance of brain tumor diagnosis can be significantly improved. In addition, through t-SNE cluster analysis and Score-Grad attention visualization, the efficient classification results of the model can be intuitively verified and explained. In conclusion, this study highlighted the importance of integrating multiple advanced technologies to extract robust deep learning features, which had important reference significance for the development of automated radiomics for brain tumors.

## Data availability statement

The original contributions presented in the study are included in the article/**Supplementary Material**. Further inquiries can be directed to the corresponding authors.

## Ethics statement

The studies involving humans were approved by the ethics committees of the First Affiliated Hospital of Yangtze University, and informed consent was waived. The studies were conducted in accordance with the local legislation and institutional requirements. Written informed consent for participation was not required from the participants or the participants’ legal guardians/next of kin in accordance with the national legislation and institutional requirements. Written informed consent was not obtained from the individual(s) for the publication of any potentially identifiable images or data included in this article because This retrospective study was approved by the ethics committees of the First Affiliated Hospital of Yangtze University, and informed consent was waived.

## Author contributions

ZW: Conceptualization, Funding acquisition, Methodology, Writing – original draft. CH: Conceptualization, Data curation, Project administration, Writing – original draft. YH: Resources, Supervision, Validation, Writing – review & editing. HL: Resources, Supervision, Validation, Writing – review & editing. CL: Formal analysis, Writing – review & editing. XW: Visualization, Writing – review & editing. YZ: Data curation, Writing – review & editing. JL: Investigation, Software, Writing – review & editing. JC: Formal analysis, Investigation, Resources, Validation, Writing – review & editing.
