# Supplementary material for: A hybrid deep learning scheme for MRI-based preliminary multiclassification diagnosis of primary brain tumors
Source: Front Oncol. 2024 Apr 30;14:1363756. doi: 10.3389/fonc.2024.1363756 (PMC11091367; doi:10.3389/fonc.2024.1363756)
Supplement: Supplementary file 1 [file Table_1.docx]

Table S1 Class-wise performance results of deep learning models in CE-MRI

| **Models** | **Label** | **AUC** | **SEN** | **SPE** | **PRE*** |
| --- | --- | --- | --- | --- | --- |
| AlexNet | 0 | 0.988 | 0.932 | 0.962 | 0.913 |
|  | 1 | 0.994 | 0.978 | 0.935 | 0.954 |
|  | 2 | 0.998 | 0.937 | 0.963 | 0.993 |
| VGG16 | 0 | 0.996 | 0.979 | 0.981 | 0.952 |
|  | 1 | 0.998 | 0.986 | 0.975 | 0.984 |
|  | 2 | 0.997 | 0.972 | 0.984 | 0.997 |
| ResNet18 | 0 | 0.998 | 0.979 | 0.978 | 0.945 |
|  | 1 | 0.999 | 0.994 | 0.965 | 0.985 |
|  | 2 | 0.999 | 0.955 | 0.989 | 0.997 |
| ResNet50 | 0 | 0.997 | 0.977 | 0.986 | 0.961 |
|  | 1 | 0.999 | 0.997 | 0.973 | 0.989 |
|  | 2 | 0.999 | 0.969 | 0.991 | 0.995 |
| DenseNet121 | 0 | 0.998 | 0.987 | 0.989 | 0.972 |
|  | 1 | 0.999 | 0.994 | 0.984 | 0.992 |
|  | 2 | 0.999 | 0.981 | 0.992 | 0.997 |
| DenseNet169 | 0 | 0.998 | 0.980 | 0.986 | 0.967 |
|  | 1 | 0.999 | 0.994 | 0.977 | 0.988 |
|  | 2 | 0.999 | 0.975 | 0.989 | 0.995 |
| GoogleNet | 0 | 0.997 | 0.960 | 0.984 | 0.957 |
|  | 1 | 0.999 | 0.990 | 0.969 | 0.983 |
|  | 2 | 0.999 | 0.975 | 0.980 | 0.988 |
| MobileNetV2 | 0 | 0.998 | 0.980 | 0.984 | 0.955 |
|  | 1 | 0.999 | 0.990 | 0.977 | 0.990 |
|  | 2 | 0.999 | 0.974 | 0.987 | 0.995 |
| MobileNetV3 | 0 | 0.997 | 0.975 | 0.983 | 0.957 |
|  | 1 | 0.999 | 0.992 | 0.971 | 0.984 |
|  | 2 | 0.999 | 0.968 | 0.986 | 0.995 |

*: PRE is the abbreviation of Precision

Table S2 Class-wise performance results of deep learning models in BT-YU

| **Models** | **Label** | **AUC** | **SEN** | **SPE** | **PRE** |
| --- | --- | --- | --- | --- | --- |
| AlexNet | 0 | 0.976 | 0.940 | 0.904 | 0.903 |
|  | 1 | 0.980 | 0.919 | 0.920 | 0.917 |
|  | 2 | 0.982 | 0.882 | 0.931 | 0.967 |
| VGG16 | 0 | 0.993 | 0.967 | 0.950 | 0.955 |
|  | 1 | 0.993 | 0.941 | 0.966 | 0.962 |
|  | 2 | 0.996 | 0.966 | 0.955 | 0.956 |
| ResNet18 | 0 | 0.989 | 0.967 | 0.937 | 0.932 |
|  | 1 | 0.989 | 0.936 | 0.957 | 0.959 |
|  | 2 | 0.993 | 0.938 | 0.953 | 0.971 |
| ResNet50 | 0 | 0.990 | 0.962 | 0.955 | 0.955 |
|  | 1 | 0.992 | 0.959 | 0.957 | 0.953 |
|  | 2 | 0.996 | 0.950 | 0.961 | 0.972 |
| DenseNet121 | 0 | 0.992 | 0.969 | 0.965 | 0.968 |
|  | 1 | 0.994 | 0.969 | 0.966 | 0.959 |
|  | 2 | 0.996 | 0.961 | 0.969 | 0.978 |
| DenseNet169 | 0 | 0.995 | 0.966 | 0.961 | 0.964 |
|  | 1 | 0.995 | 0.962 | 0.964 | 0.949 |
|  | 2 | 0.998 | 0.960 | 0.964 | 0.985 |
| GoogleNet | 0 | 0.989 | 0.962 | 0.943 | 0.939 |
|  | 1 | 0.991 | 0.951 | 0.951 | 0.954 |
|  | 2 | 0.995 | 0.933 | 0.957 | 0.973 |
| MobileNetV2 | 0 | 0.993 | 0.965 | 0.947 | 0.948 |
|  | 1 | 0.993 | 0.954 | 0.956 | 0.952 |
|  | 2 | 0.996 | 0.938 | 0.960 | 0.974 |
| MobileNetV3 | 0 | 0.985 | 0.945 | 0.925 | 0.928 |
|  | 1 | 0.985 | 0.937 | 0.932 | 0.920 |
|  | 2 | 0.986 | 0.910 | 0.941 | 0.971 |

Table S3 Class-wise performance results of machine learning models in CE-MRI

| **Models** | **Label** | **AUC** | **SEN** | **SPE** | **PRE** |
| --- | --- | --- | --- | --- | --- |
| LR | 0 | 0.991 | 0.965 | 0.994 | 0.978 |
|  | 1 | 1.000 | 0.997 | 0.979 | 0.997 |
|  | 2 | 0.997 | 0.989 | 0.986 | 0.979 |
| NaiveBayes | 0 | 0.539 | 0.078 | 0.998 | 1.000 |
|  | 1 | 0.997 | 0.997 | 0.602 | 0.997 |
|  | 2 | 0.847 | 1.000 | 0.693 | 0.589 |
| SVM | 0 | 0.992 | 0.965 | 0.994 | 0.978 |
|  | 1 | 0.999 | 0.997 | 0.979 | 0.997 |
|  | 2 | 0.997 | 0.989 | 0.986 | 0.979 |
| RandomForest | 0 | 0.990 | 0.957 | 0.989 | 0.964 |
|  | 1 | 0.998 | 0.993 | 0.973 | 0.993 |
|  | 2 | 0.995 | 0.984 | 0.981 | 0.979 |
| ExtraTrees | 0 | 0.990 | 0.965 | 0.992 | 0.971 |
|  | 1 | 0.998 | 0.997 | 0.976 | 0.997 |
|  | 2 | 0.995 | 0.984 | 0.986 | 0.979 |
| XGBoost | 0 | 0.987 | 0.950 | 0.996 | 0.985 |
|  | 1 | 0.998 | 0.997 | 0.976 | 0.990 |
|  | 2 | 0.994 | 0.995 | 0.981 | 0.979 |
| LightGBM | 0 | 0.987 | 0.965 | 0.996 | 0.986 |
|  | 1 | 0.998 | 0.997 | 0.982 | 0.997 |
|  | 2 | 0.994 | 0.995 | 0.986 | 0.979 |
| AdaBoost | 0 | 0.992 | 0.965 | 0.994 | 0.978 |
|  | 1 | 0.999 | 0.993 | 0.982 | 0.997 |
|  | 2 | 0.994 | 0.995 | 0.984 | 0.979 |
| MLP | 0 | 0.992 | 0.965 | 0.989 | 0.965 |
|  | 1 | 1.000 | 0.993 | 0.976 | 0.997 |
|  | 2 | 0.997 | 0.984 | 0.984 | 0.979 |

Table S4 Class-wise performance results of machine learning models in BT-YU

| **Models** | **Label** | **AUC** | **SEN** | **SPE** | **PRE** |
| --- | --- | --- | --- | --- | --- |
| LR | 0 | 0.995 | 0.981 | 0.980 | 0.977 |
|  | 1 | 0.994 | 0.970 | 0.986 | 0.975 |
|  | 2 | 1.000 | 1.000 | 0.976 | 1.000 |
| NaiveBayes | 0 | 0.978 | 0.977 | 0.980 | 0.977 |
|  | 1 | 0.978 | 0.970 | 0.984 | 0.975 |
|  | 2 | 0.999 | 1.000 | 0.974 | 0.991 |
| SVM | 0 | 0.990 | 0.981 | 0.980 | 0.977 |
|  | 1 | 0.985 | 0.970 | 0.986 | 0.980 |
|  | 2 | 1.000 | 1.000 | 0.976 | 0.991 |
| RandomForest | 0 | 0.984 | 0.981 | 0.980 | 0.977 |
|  | 1 | 0.984 | 0.970 | 0.986 | 0.975 |
|  | 2 | 1.000 | 1.000 | 0.976 | 1.000 |
| ExtraTrees | 0 | 0.989 | 0.984 | 0.980 | 0.977 |
|  | 1 | 0.986 | 0.970 | 0.989 | 0.980 |
|  | 2 | 1.000 | 1.000 | 0.978 | 1.000 |
| XGBoost | 0 | 0.982 | 0.988 | 0.977 | 0.973 |
|  | 1 | 0.976 | 0.965 | 0.992 | 0.985 |
|  | 2 | 1.000 | 1.000 | 0.978 | 1.000 |
| LightGBM | 0 | 0.989 | 0.996 | 0.974 | 0.970 |
|  | 1 | 0.982 | 0.959 | 0.997 | 0.995 |
|  | 2 | 1.000 | 1.000 | 0.980 | 1.000 |
| AdaBoost | 0 | 0.987 | 0.984 | 0.980 | 0.977 |
|  | 1 | 0.986 | 0.970 | 0.989 | 0.980 |
|  | 2 | 1.000 | 1.000 | 0.978 | 1.000 |
| MLP | 0 | 0.994 | 0.984 | 0.980 | 0.977 |
|  | 1 | 0.994 | 0.970 | 0.989 | 0.980 |
|  | 2 | 1.000 | 1.000 | 0.978 | 1.000 |
